# Differences in Children’s Food and Beverage Consumption between School and Summer: Three-year findings from the What’s UP (Undermining Prevention) with Summer Observational Cohort Study

**DOI:** 10.21203/rs.3.rs-6255625/v1

**Published:** 2025-05-07

**Authors:** Michael W. Beets, Sarah Burkart, Christopher D. Pfledderer, Elizabeth Adams, R. Glenn Weaver, Bridget Armstrong, Keith Brazendale, Xuanxuan Zhu, Alexander McLain, Gabrielle Turner-McGrievy, Russell Pate, Andrew Kaczynski, Amanda Fairchild, Brian Saelens, Hannah Parker, Amy L. Yaroch, Emily Eglitis, Anthony J. Holmes

**Affiliations:** University of South Carolina; University of South Carolina; UTHealth Houston; University of South Carolina; University of South Carolina; University of South Carolina; University of Central Florida; University of South Carolina; University of South Carolina; University of South Carolina; University of South Carolina; University of South Carolina; University of South Carolina; Seattle Children’s Hospital; University of South Carolina; Center for Nutrition and Health Impact; University of South Australia; University of South Carolina

**Keywords:** Diet, Vacation, Children, Poverty, Low-Income

## Abstract

**Background:**

Summer vacation is a time when youth gain excessive weight. A key driver of unhealthy weight gain is poor dietary quality. The absence of consistent structure (e.g., school), is hypothesized to be one of the reasons for lower diet quality during summer. This study examined differences in school and summer dietary quality among a diverse cohort of children across three years. We also examined the impact of attending structured programs on children’s diets.

**Methods:**

Parents of 1,298 children (age 5–14 years, 48% girls) completed a time use diary each day for 14-days during school (April/May) and again in summer (July) from 2021 to 2023, for a total of 6 timepoints. The daily diary collected information on the child’s location and dietary intake for that day. Mixed-effects models examined the odds (OR) of consuming a food/beverage (e.g., fruit, vegetable, soda, salty snacks) on a given day during school vs. summer, overall and by income. Models also examined the impact of attending structured programming during summer (e.g., summer day camp) on the likelihood of consumption.

**Results:**

A total of 39,983 time use diaries were completed. Overall, children were less likely to consume fruit, vegetables, milk, 100% juice, and salty snacks (OR range 0.63 to 0.87), and they were more likely to consume non-carbonated sweetened beverages, soda, frozen desserts, and fast food (OR range 1.17 to 1.63) during the summer compared to school. On summer days with structured programming, children were more likely to consume fruits, vegetables, milk, salty snacks, sweetened beverages (OR range 1.13 to 1.45), and they were less likely to consume frozen desserts, fast food, and soda (OR range 0.63 to 0.90). Few differences were observed between income groups.

**Conclusions:**

Children were less likely to report consumption of more healthful foods/beverages and more likely to report consumption of less healthful foods/beverages during summer compared to school. Attending structured programming during summer is associated with improved diet – suggesting such settings have potential to modify dietary intake.

## Background

Substantial evidence demonstrates children are vulnerable to excessive weight gain during the summer months as compared to the school year.^1–21^ One factor contributing to unhealthy weight gain during summer is changes in dietary intake. While some studies^22–25^ indicate healthful foods (e.g., fruits and vegetables) are consumed less and unhealthful foods/beverages (e.g., added sugars) are consumed more during summer, recent reviews indicate there is inconsistent evidence demonstrating diet is worse in summer for children.^26,27^

Why children’s diets may be more healthful during school and less healthful during summer is multifaceted. A leading perspective to understand these differences is the Structured Days Hypothesis (SDH).^28,29^ The SDH posits children’s dietary intake during school is influenced by the consistent, structured environment schools provide to children. For schools in the United States (U.S.), schools are required to adhere to federal nutrition guidelines for meals (breakfasts, lunch, dinners) and snacks.^30–32^ Consuming school meals is associated with better overall dietary intake.^30,33–36^ During the school week (M-F) children consume a large proportion of their daily caloric intake from foods/beverages served at school and eating opportunities at school are consistent and regulated (i.e., recess, lunch). Although schools allow foods/beverages to be brought from home, a large portion of children consume the foods/beverages provided by a school^37–39^ and the meals provided by school are generally more healthful than those brought from home (e.g., lunch provided by school vs. lunch brought from home).^40–42^ Consumption of school meals and the introduction of school meal policies (e.g., Healthy, Hunger-Free Kids Act of 2010) is associated with reductions in zBMI.^36^ Evidence suggests foods/beverages consumed outside of school offset the healthfulness of foods/beverages consumed at school for children’s overall dietary intake.^43^ Given the home environment may not promote optimal dietary intake, especially in families from low-income households, consuming a large portion of one’s foods/beverages in school can serve as an important contributor to overall improved dietary intake and maintenance of healthy weight.

During the summer, the 5 day a week presence of school is removed, leaving children with greater exposure to, and reliance upon, the food available at home. While summer feeding programs (e.g., Summer Food Service Program, Seamless Summer Option) exist to minimize this decline in federally funded meals during the summer months, their reach is significantly limited and often results in limited food access. Thus, the removal of the consistent structure of school and the overarching guidelines for foods/beverages readily available to them in this setting could lead children to consume more unhealthful foods/beverages during summer and can be susceptible to more, unsupervised eating opportunities.^44^ However, some children spend time in structured settings during the summer, namely some form of summer programming (e.g., summer school, summer day camps). Such programs often participate in summer feeding programs and thus adhere to federal reimbursement guidelines that govern the types of foods/beverages served. Regular exposure to such settings could lead to improved dietary intake during the summer.^9,45–48^ However, not all youth have equal access to summer programming. National data indicate the majority of children enrolled in summer programming are White and from higher income households.^49,50^ Disproportionate access to summer programming could lead to greater exposure to the home food environment for children from lower-income households who often experience financial barriers to affording nutrient-rich meals. Extended exposure to the home food environment at a greater frequency during summer could be a primary driver of unhealthy weight gains observed during this time. The purpose of this study was to examine changes in children’s diet from school and summer and to explore the role of attending structured programming on diet quality in the summer.

According to the SDH, we hypothesized the following associations: a) Children would be more likely to consume less healthful foods/beverages during summer and more likely to consume unhealthful foods/beverages, as compared to the school year; b) During the 7-day week, children would be more likely to consume less healthful foods/beverages and more likely to consume unhealthful foods/beverages on the weekends, compared to weekdays, during school and summer; and c) During the summer, days where children attended a summer program would be associated with a greater likelihood of consuming healthful foods/beverages and a lower likelihood of consuming unhealthful foods beverages, compared to days where children do not attend a summer program.

## Methods

### Study Design and Sample.

This study used data from the What’s UP (Undermining Prevention) with Summer study (National Institutes of Health R01DK116665, WUP) designed to understand summer effects on unhealthy weight gain among elementary-aged children in the United States. The design was a longitudinal observational cohort that followed elementary-aged children (5–12 years) across three years (2021, 2022, and 2023) and completed measures during school (April/May) and summer (July) each year. Children were recruited from 17 elementary schools in a mid-sized metropolitan area in the southeastern United States located at 34°N. Invitations to participate in the study were provided to parents via study flyers with a QR code and distributed via school texting services (e.g., Class Dojo). The QR code was linked to an online HIPAA compliant website to provide electronic consent. Children of parents who provided online consent, then provided verbal assent during in-person assessments. Parents and children had the right to discontinue participation in the study at any time and were asked each year if they wanted to continue in the study. Informed consent was obtained from all the participants and/or their legal guardians. The reporting of this study conforms to the STROBE checklist.^51^ This study was approved by the lead author’s Institutional Review Board (Pro00080382).

### Child and Household Characteristics.

At the school measurement timepoint each year (April/May), parents completed an online survey via their smartphone about the demographics of their child (biological sex and age), information about the total annual household income, and the number of people (adults and children) living in their home. This information was used to calculate the ratio of poverty to income according to U.S. Federal Poverty Guidelines established by the Department of Health and Human Services.^52^

### Refreshing Longitudinal Cohort.

To account for parents/children electing to discontinue in the study, we refreshed the sample^53–55^ in the early spring (February/March), prior to school data collection in April/May each year. Refreshing the sample replaces students who dropped out of the study with students who had similar demographic characteristics. Refreshing is methodology consistent with other large-scale cohort studies.^54–56^ Criteria of the refreshment sample was to ensure similar proportions of students were present in the study based on child sociodemographics (i.e., biological sex, race/ethnicity, household income) from the initial year of data collection in 2021 and to replace children based upon the grade as the cohort aged over time.

### Dietary Measure.

The Dietary Screener Questionnaire from the Healthy Communities Study (HCS)^57^ was used to collect dietary data on children enrolled in What’s UP. The screener consisted of 23 items from the HCS that assessed children’s intake of fruits and vegetables; dairy; sugar-sweetened beverages; other energy-dense foods of minimal nutritional value (e.g., chocolate/candy, donuts/sweet rolls, cookies/cakes/pies, ice cream/frozen desserts, chips/crackers); and fast food. Parents, along with their child, completed the screener at the end of each day, for a total of 14-days, during school (April/May) and again for 14-days in summer (July), reporting on what their child ate and/or drank that day. Unlike the original screener, which asked respondents about consumption over the past 30 days (e.g., 1 time last month, 2 times per week), only response options referring to the frequency of consumption for that day were included (e.g., 1 time today, 2–3 times today). Parents and their child completed the screener as part of the time use record (described in detail below).

### Time Use Measure.

The time use record was developed in Qualtrics and distributed to parents at 8PM each evening of the 14-day measurement period during school and summer to complete on their smartphone. The time use record was based on the day reconstruction method.^58^ The time use record involved parents completing a series of questions about their child’s previous 24hrs. The first question asked about the time their child went to bed the previous night, followed by when their child woke today. The next series of questions asked about whether their child left where they were (e.g., woke up at home, did child leave home today?) and if “yes”, where their child went and at what time (e.g., left home and went to school at 7:15AM). Parents reported time in 15min increments (e.g., 9:00AM, 9:15AM, 9:30AM…). This sequence of questions continued until parents reported the entire day with where their child was and at what times. The time use record provided a list of common locations where a child may be that a parent could select from: home, before school program, school, afterschool program, sports, music/theater/dance, neighborhood pool/water, park/river/lake/beach, park/playground, employment/volunteer, church/synagogue/mosque or other religious activity, friend’s house, relative’s house, restaurant/fast food outlet, errands/shopping/appointments, tagging along/attending family or friend’s event/activity, or summer day camp. There was an additional “other” option for parents to write a specific activity/setting not represented. For the purpose of this study, we classified sports, music/theater/dance, employment/volunteer, places of worship, summer day camps, and summer learning programs as structured settings in the summer. Embedded at the end of the time use record was the dietary screener. Each time use record took an average of 6min to complete (median 4.8min, IQR 3.5 to 7.1min).

### Statistical Analysis.

The primary analysis compared differences in children’s odds of consuming different food/beverage categories on a given day between school and summer. After review of the distribution of the responses, items were dichotomized into “did not eat/drink today” versus “ate/drank today”. Items were collapsed into the following categories for analyses: fruit, vegetables, 100% fruit juice, milk (unflavored and flavored milk), frozen desserts, other desserts (pastries/doughnuts and cookies cakes, pies), non-carbonated sugar-sweetened beverages (non-100% fruit juice, energy drinks, flavored water, sports drinks, coffee drinks), sugar-sweetened and diet soda/pop, and salty snacks (chips/pretzels). A series of multi-level, generalized linear models that accounted for the nesting of days within children, within families, within schools, were examined for each food/beverage dependent variable with the following contrasts of interest. For our main analysis we examined differences in the probability of consuming a food/beverage on a given day between school and summer. Analyses also examined changes in the probability of consuming a food/beverage on a given day between school and summer by each of the 3 poverty to income ratio groups (insert info about 3 groups). To examine the association of exposure to structured programming and dietary intake in the summer, we used the exposure to structure data from the time use record and created two exposure metrics: 1) dichotomized exposure to structure or not on a given day; and 2) exposure to structure in number of minutes per day and categorized this into no (reference group), low (30–60min/d), moderate (> 60, < 200min/d) and high (> 200min/d) of structured programming attended. All models included biological sex, age, parent education, poverty to income ratio, food insecurity status, and use of either the Supplemental Nutrition Assistance Program (SNAP) or Women, Infants, and Children (WIC) program as covariates.

## Results

The number of children by age in years and daily diaries across the 6 assessments are presented in Table 1. Across the 3-year study, a total of 1,298 children completed an average of 31 days of time diaries each across the 6 measurement occasions. The demographics of the cohort across each assessment period are presented in Table 2.

### School vs. Summer.

**Figure 1:**
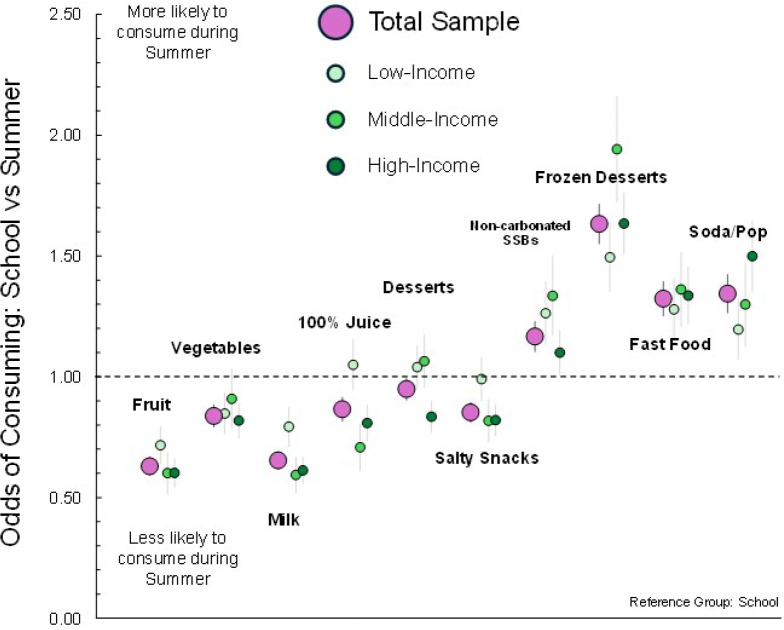
Odds of consuming a food or beverage during the summer, compared to when children are in school

The comparison between foods/beverages consumed during school compared to summer can be found in Fig. 1 and **supplemental Table 1**. For the entire sample and by each of the three income-subgroups, children were, in general, more likely to consume unhealthful foods/beverages (e.g., frozen desserts, soda/pop) and less likely to consume healthful foods/beverages (e.g., fruits, dairy) during the summer in comparison to when they were in school. However, during summer, children tended to consume fewer non-frozen desserts and salty snacks (overall sample and high-income group). Of the 40 comparisons, 30 supported the SDH. Of the remaining 10, 5 did not support the SDH (i.e., in the opposite direction of the SDH) and 5 were not statistically significant.

### Weekdays vs. Weekend Days: School and Summer, separately.

**Figure 2:**
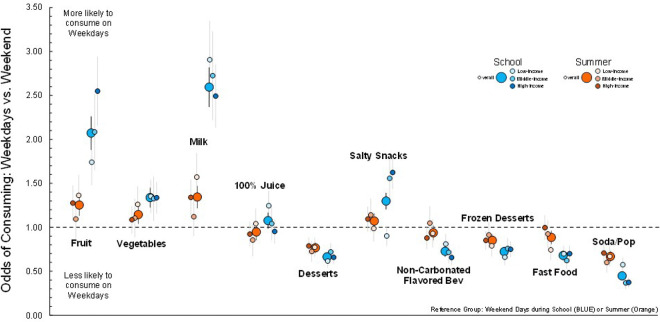
Odds of consuming a food or beverage on a weekdays, compared to weekend days, during school (blue) and summer (orange), separately

For school timepoints, in the entire sample children were more likely to consume healthful foods/beverages (e.g., fruit, dairy) and less likely to consume unhealthful foods (e.g., fast food, soda/pop) on weekdays (M-F) compared to weekend days (Sat-Sun) – see Fig. 2 and **supplemental Table 1**. Subgroup analysis by income demonstrated the same pattern. During weekdays in the overall sample and children in the middle- and high-income groups were more likely to consume salty snacks compared to the weekend days, whereas children from low-income households were less likely to consume salty snacks.

During the summer, similar associations were observed, with more healthful dietary patterns during weekdays vs weekend days. However, these associations tended to be smaller in magnitude compared to weekday vs weekend day comparisons during school - for example OR 2.07 school compared to OR 1.25 summer for weekdays vs weekend days for fruit consumption. Separate comparisons across income groups indicated children in the low- and high-income groups exhibited more healthful dietary patterns on summer weekdays vs weekend days, while children from middle-income households had more similar dietary intakes on weekdays and weekend days in the summer.

### Structure during the Summer.

**Figure 3:**
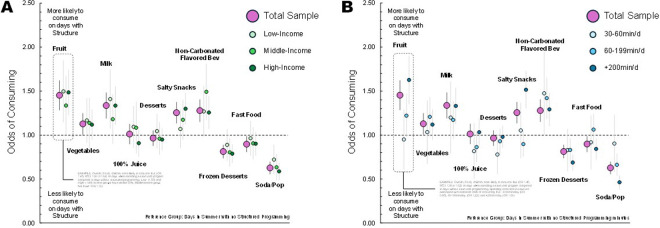
Association of days spent in structure to the odds of consuming a food or drink on that day during summer. A: Odds of consuming on a given day when attending any structure (binary) during summer B: Odds of consuming for days when attending structure based on time (minutes) during summer

In the summer, on days when children attended some form of structured programming (binary variable attended vs did not attend), they tended to exhibit more healthful dietary patterns compared to days without attending structured programming (Fig. 3A and **supplemental Table 1**). This pattern was similar for each income subgroup. When examining exposure to structure based on minutes spent in a structured setting each day, a dose response association emerged (Fig. 3B and **supplemental Table 1**). As children spent more time in a structured setting in the summer, their dietary pattern improved. The greatest improvements were observed for children spending 200 or minutes per day in a structured setting in the summer.

## Discussion

Summer is increasingly being recognized as a period of time where children are at a greater risk for unhealthy weight gain.^1–21^ Generally, it is well established that diet is a leading behavioral contributor to unhealthy weight gains in children, however, evidence regarding whether children’s dietary patterns during the summer are different than their dietary intake during school are mixed.^26,27^ According to the SDH, children should consume more unhealthful foods during summer, compared to school, given 1) limited access to federally funded meals that adhere to nutritional guidelines in the summer, and 2) increased freedoms (e.g., more snacking opportunities) that may operate during summer when days may be less-structured with the removal of school. The findings in this study indicate children exhibit more unhealthy dietary patterns during summer, compared to school, and on weekend days, compared to weekdays, both during school and summer. Importantly, during the summer, attending structured programming was associated with a more healthful dietary pattern, compared to days without structure. Overall, these findings have important implications for establishing summer as time with increased risk for unhealthful dietary intake, as well as providing additional theoretical support for the SDH of the importance of structured programming (school and summer programs) on beneficially influencing children’s dietary intake.

The main findings were that children were more likely to consume unhealthful foods during the summer, compared to school. These effects were consistent across income groups, indicating summer had a similar effect on dietary intake regardless of socioeconomic status. This shows schools provide a seemingly equitable impact on child diet intake and conversely, when children are removed from the almost daily exposure to the school environment and with that the nutrition regulations that govern the nutritional quality of foods/beverages readily available, they are at a greater risk of consuming less healthful foods/beverages and consuming more unhealthful foods/beverages.^31,35,36,38,39,41–43^ The importance of the school food environment is further highlighted in the comparison of consumption between weekdays and weekend days where the largest odds were observed for school days vs weekend days when children were in school.

A similar, albeit smaller, effect was observed for summer weekdays vs weekend days. Analyses indicated on days when children attended a summer program (e.g., day camp), they were more likely to consume healthful foods/beverages and less likely to consume unhealthful foods/beverages. When considering the amount of time spent at a summer program, the longer attendance at a summer program during a day was associated with a greater odds of consuming healthful foods/beverages and a lower odds of consuming unhealthful foods beverages. These findings from summer, along with those during the school year, although observational, provide empirical support for the impact of spending time in a structured setting and the association with improved dietary behaviors.^26,28^

While most associations were in the direction hypothesized by the SDH, there were several food/beverage items that demonstrated associations in the opposite direction. Specifically, in the summer, attending a summer program was associated with an increased odds of consuming salty snacks and non-carbonated flavored beverages. A plausible reason for these associations could be salty snacks are items that are reimbursable via federal nutrition programs, such as the Summer Food Service Program (https://www.fns.usda.gov/summer/sunmeals). Thus, salty snacks would likely be served on a regular basis in summer programs, leading to an increase in their consumption for children attending. Secondly, during the summer, due to temperature and humidity, there is an added need to ensure appropriate hydration. With this, children bring sports drinks and other flavored beverages when attending a summer program.^59–61^ These factors could explain why attending a summer program was associated with increased odds of consuming these items.

There are a number of strengths to this study. These include the large and diverse sample of children participating, the longitudinal design, the assessment of diet during school and summer, and the large number of days collected during both school and summer. There are several weaknesses. One is the use of a dietary screener to measure dietary intake. While this measure is not as comprehensive as a 24hr dietary recall, this tool facilitated a quick and lower-burden assessment of dietary intake on a daily basis, which in turn, allowed for the comparison of weekdays vs weekend days, as well as days during the summer where children did and did not attend a summer program. This tool also limited the ability to quantify the amount consumed and reduced our ability to quantify macro- and micro-nutrients. Another weakness is the limited geographical location where these data were collected. Summer at other latitudes would be associated with varying temperatures, thereby changing the experiences of children during this time. Nevertheless, this study was conducted in the southeastern United States which has among the highest rates of obesity and obesogenic behaviors^62^ and thus, the findings from this study, at minimum, generalize to this geographical area.

In summary, children were more likely to consume unhealthful foods/beverages and less likely to consume healthful foods/beverages during summer, compared to when they are in school. Evidence of the importance of structured environments was observed when examining weekdays vs weekend days during school and summer, separately. Importantly, we observed an association between attending summer programming and consuming more healthful and less healthful foods/beverages during the summer, suggesting such programming during the summer could play an important role in promoting a healthful diet during the summer.

## Figures and Tables

**Table 1:** Number of children and daily diaries by assessment year.

		Number of Children by Age (years)										
Year	Timepoint	5	6	7	8	9	10	11	12	13	14	Total
2021	School	9	69	64	102	123	138	70	19			**594**
	Summer	6	44	68	79	94	116	74	25			**506**
2022	School	3	29	78	85	109	121	137	59	14		**635**
	Summer		22	54	66	91	85	112	63	18		**511**
2023	School		1	26	63	73	148	177	150	58	11	**707**
	Summer			20	51	68	117	152	146	73	15	**642**
	Total	**18**	**185**	**341**	**463**	**607**	**760**	**716**	**389**	**105**	**11**	
		Number of Daily Diaries by Age (years)										
Year	Timepoint	5	6	7	8	9	10	11	12	13	14	Total
2021	School	109	742	699	1130	1415	1553	778	207			**6633**
	Summer	67	514	733	899	1083	1340	818	309			**5763**
2022	School	34	339	812	847	1137	1329	1436	600	160		**6694**
	Summer		214	546	726	922	866	1206	671	182		**5333**
2023	School		14	298	714	844	1723	2029	1749	691	135	**8197**
	Summer			230	557	796	1313	1732	1656	885	194	**7363**
	Total	**210**	**1823**	**3318**	**4873**	**6197**	**8124**	**7999**	**5192**	**1918**	**329**	

Timepoint: School = April/May; Summer = July

**Table 2:** Child demographics by assessment period

	2021				2022				2023			
	School		Summer		School		Summer		School		Summer	
Sample (N)	594		506		635		511		707		642	
Age (year, M, SD)	8.8	± 1.7	8.9	± 1.7	9.5	± 1.8	9.7	± 1.8	10.6	± 1.6	10.8	± 1.6
Females (%)	48%	± 0.5	48%	± 0.5	49%	± 0.5	49%	± 0.5	47%	± 0.5	47%	± 0.5
Race/Ethnicity (%)												
Black	35%		33%		44%		42%		38%		38%	
White	57%		60%		45%		48%		53%		54%	
Other	3%		3%		3%		3%		2%		2%	
Hispanic	6%		5%		8%		8%		7%		7%	
Poverty to Income Ratio (M, SD)	2.6	± 1.1	2.7	± 1.1	2.3	± 1.1	2.4	± 1.0	2.4	± 1.1	2.5	± 1.0
200% or Below Pov (%)	35%		33%		38%		35%		36%		34%	
Food Insecure (%)	14%	± 0.4	16%	± 0.4	19%	± 0.4	19%	± 0.4	22%	± 0.4	21%	± 0.4
Supplemental Nutrition Assistance Program (%)	9%	± 0.3	10%	± 0.3	12%	± 0.3	10%	± 0.3	10%	± 0.3	9%	± 0.3
Women, Infants, and Children (%)	5%	± 0.2	6%	± 0.2	4%	± 0.2	3%	± 0.2	3%	± 0.2	3%	± 0.2
Avg. Number of Time Use Diaries (M, SD)	12.4	± 2.7	12.4	± 2.5	11.9	± 2.7	11.5	± 2.4	12.7	± 2.4	12.5	± 2.4
Avg. Days with Structure Reported (M, SD)			2.9	± 3.1			3.9	± 3.3			2.9	± 3.2
Avg. Minutes per day of Structure during Summer (M, SD)			59.0	± 153.3			61.2	± 152.9			65.4	± 152.9
Avg. Minutes per day of Structure for Children Reporting going to Structure during Summer (M, SD) (does not include ZERO structure)			321.2	± 209.5			298.6	± 208.1			308.2	± 187.9

## Data Availability

The datasets generated and/or analyzed in the current study are not publicly available because additional analyses are ongoing. Data will be made available from the corresponding author on reasonable request at the time the primary outcome papers are published.
